# The future of ECG monitoring – can psychiatry take the ‘lead’?

**DOI:** 10.1192/bjb.2023.7

**Published:** 2023-04

**Authors:** Jack Mumford, Eimear Devlin, George Crowther

**Affiliations:** Liaison Psychiatry Specialty Doctor, Leeds and York Partnership NHS Foundation Trust, UK. Email: jack.mumford@nhs.net; ST4 Old Age Psychiatry Humber Teaching NHS Foundation Trust, UK; Consultant Old Age Liaison Psychiatry, Leeds and York Partnership NHS Foundation Trust. Visiting Senior Lecturer, University of Leeds.

Electrocardiogram (ECG) monitoring is an essential part of safe prescribing in psychiatry, especially when a patient is admitted to an acute in-patient ward in England. Any psychiatrist who has worked on such a ward will appreciate the challenges that come with completing this ‘simple’ investigation.

Despite it being almost 100 years since Willem Einthoven was awarded the Nobel prize ‘for the discovery of the mechanism of the electrocardiogram’ and almost 70 years since the American Heart Association published their recommendation for standardisation of 12-lead electrocardiogram, there have been few recent practical changes in the way ECGs are obtained in UK clinical practice.

Although 12-lead ECG machines have become smaller and more advanced, patients are still required to expose their chests; allow for all ten physical leads to be attached, often using uncomfortable or irritating stickers and clips; and lie still for several seconds while a reading is taken.

We recently completed a regional service evaluation across Yorkshire and the Humber examining ECG compliance on adult, older adult and forensic wards. Our data, gathered from 529 patients across 25 wards, demonstrated that only 82% (*n* = 432) of patients had an ECG at any point during admission, of which only 63% (*n* = 272) were completed in 24 h. Concerningly, among the patients taking antipsychotics (*n* = 378), these numbers were lower–80% (*n* = 303) and 50% (*n* = 188), respectively.

Qualitative analysis of results demonstrated that the most common reasons for not having an ECG completed were ‘patient-related factors’. Where a specific reason was given, the most common were ‘aggressive, agitated, anxious, paranoid’ (159 of 257; 62%). ECGs can appear threatening and intrusive; clothes on the upper body need to be removed, body hair may need to be shaved and the chest leads can look frightening. When someone is already distressed, this fear is likely to be exaggerated. Anxiety, past physical and sexual abuse, or gender identity concerns may further exacerbate this.

ECG monitoring in psychiatry is safety driven and exists because antipsychotics (particularly at high doses),^1^ some antidepressants and methadone can predispose to life-threatening heart arrhythmias.^2,3^ Examples include QTc prolongation, PR interval prolongation and, in extremis, torsade de pointes.^1^ ECGs should be conducted as soon after admission as possible, preferably within the first 24 h.^4^ Our results show that patients are therefore at risk of potentially fatal side-effects, and prescribers fall short of evidence-based and guideline-directed practice.^1–4^

Improving compliance with ECG monitoring, particularly in those prescribed pro-arrhythmic drugs, is a patient safety priority. Educating patients and mental health professionals about the need for ECGs is important, but reducing the intrusiveness and increasing the accessibility of ECG monitoring is called for. Technological advancements are being embraced in other areas of psychiatric practice; why not extend this to ECG monitoring? The advent of handheld ECG machines could be a solution to ensure all patients receive high-quality safe healthcare. These devices are yet to be approved in the UK for measuring QTc, but early validation work seems positive.^5^
